# Tuberculosis Osteomyelitis as an Indolent Bone Mass

**DOI:** 10.7759/cureus.42849

**Published:** 2023-08-02

**Authors:** Brandon W Knopp, Payton Yerke Hansen, Kimberlee Persaud, Robert Reid

**Affiliations:** 1 Medical Education, Florida Atlantic University Charles E. Schmidt College of Medicine, Boca Raton, USA; 2 Pediatrics, Joe DiMaggio Children's Hospital, Hollywood, USA; 3 Pediatric Infectious Diseases, Joe DiMaggio Children's Hospital, Hollywood, USA

**Keywords:** anti-tuberculosis treatment, brodie's abscess, pediatric infectious disease, pediatrics, bone mass, osteomyelitis, tuberculosis osteomyelitis, tuberculosis

## Abstract

Tuberculosis (TB) is a pulmonary disease with potential extrapulmonary manifestations that is caused by the bacteria *Mycobacterium tuberculosis*. Despite advancements in treatment, TB remains a worldwide public health concern. TB osteomyelitis accounts for approximately 3-5% of all extrapulmonary TB cases. We present a case of humeral TB osteomyelitis in a 22-month-old female with no pulmonary or systemic symptoms. This case offers insight into the diagnosis and management of TB osteomyelitis. A 22-month-old previously healthy Haitian-American female presented with a one-month history of a palpable mass over the anterolateral aspect of the proximal humerus without overlying erythema or soft tissue swelling. No additional symptoms were reported. She had no recent sick contacts but had visited Haiti during her infancy. Right proximal humerus X-ray and subsequent MRI revealed proximal humeral osteomyelitis with an intraosseous Brodie's abscess. Incision and drainage extracted caseous material, which tested positive for *Mycobacterium tuberculosis* via a polymerase chain reaction. The diagnosis was confirmed via positive QuantiFERON-TB Gold and purified protein derivative testing. The patient was treated with levofloxacin, isoniazid, pyrazinamide, pyridoxine, and rifampin during the hospitalization. Following discharge, she was continued on her antibiotic regimen and managed by the Florida Department of Health. Post-discharge, X-rays at three and twelve months showed evidence of lesion healing. TB osteomyelitis is a rare manifestation of TB infection, which may present insidiously without systemic or pulmonary symptoms. As timely treatment is vital to preventing complications, bone masses of unknown etiology should be investigated for TB infection, even without additional symptoms.

## Introduction

Despite advancements in treatment, tuberculosis (TB) remains a worldwide public health concern, with cases reported in every country. Many low- and middle-income countries have a notable incidence of TB; however, 87% of new TB cases occur in the 30 recognized high-TB-burden countries [1,2]. The World Health Organization (WHO) estimates that in 2021 more than 10.6 million people were diagnosed with active or latent TB globally, including 1.2 million children. In the United States, the number of active TB cases has been gradually dropping for decades, with 16,306 cases reported in 2000 [3]. Notably, the TB incidence in the United States increased slightly from 7,874 cases reported in 2021 to 8,300 reported TB cases in 2022 [4]. 

TB is primarily a pulmonary disease that frequently manifests with symptoms including fever, night sweats, weight loss, cough, hemoptysis, and chest pain [2,5]. However, TB can also disseminate hematogenously to almost any organ, with extrapulmonary spread in about 14% of cases [6]. TB osteomyelitis accounts for approximately 3-5% of all extrapulmonary TB cases [7]. The most common sites of TB osteomyelitis include the spine, small bones of the hands and feet, femur, and tibia [8,9]. Though most cases have pulmonary involvement, nearly 20% of TB cases have no pulmonary manifestations [10]. Extrapulmonary TB is difficult to diagnose in cases with no pulmonary manifestations, particularly in children [2]. As delays in treatment can be detrimental to the patient's health due to an increased risk of local and distal disease spread, growing awareness for extrapulmonary TB manifestations is vital to patient care. Likewise, early recognition can limit the spread to healthcare workers and the community. Herein, we present a case of humeral TB osteomyelitis in a 22-month-old female presenting as an indolent bone mass without systemic manifestations.

## Case presentation

A 22-month-old previously healthy Haitian-American female presented with a one-month history of a palpable mass on her right shoulder. She had no history of illness, cough, hemoptysis, shortness of breath, fever, soft tissue swelling, shoulder pain, weight loss, night sweats, or changes in appetite. Notably, the patient had fallen off a bed and landed on her right shoulder a month before the parents noticed the mass. The patient had limited use of her arm immediately following the fall; however, the symptoms resolved within a day and there were no residual deficits. The father denied any recent history of difficulty moving the arm or signs of pain when the patient moved her arm. Significant travel history included a six-month visit to Haiti beginning at one month of age. The parents reported no sick contacts or international visitors since visiting Haiti. There were no reported sick contacts in Haiti or since returning home. No international visitors were reported. The patient was admitted for further evaluation and management. Pediatric orthopedics, interventional radiology, and infectious disease specialists were consulted.

Physical exam

The patient appeared healthy, alert, and appropriately interactive. She presented without fever and with normal vital signs. Her physical examination was notable for a mass over the anterolateral aspect of the proximal humerus without overlying erythema or soft tissue swelling. The mass was hard and fixed. She had regular physical activity, normal passive and active range of motion in her right arm, and no tenderness to palpation. No rashes or enlarged lymph nodes were appreciated. All other physical exam findings were normal. Laboratory tests including complete blood count, C-reactive protein, comprehensive metabolic panel, and erythrocyte sedimentation rate were within normal limits.

Imaging studies and procedures

**Figure 1:**
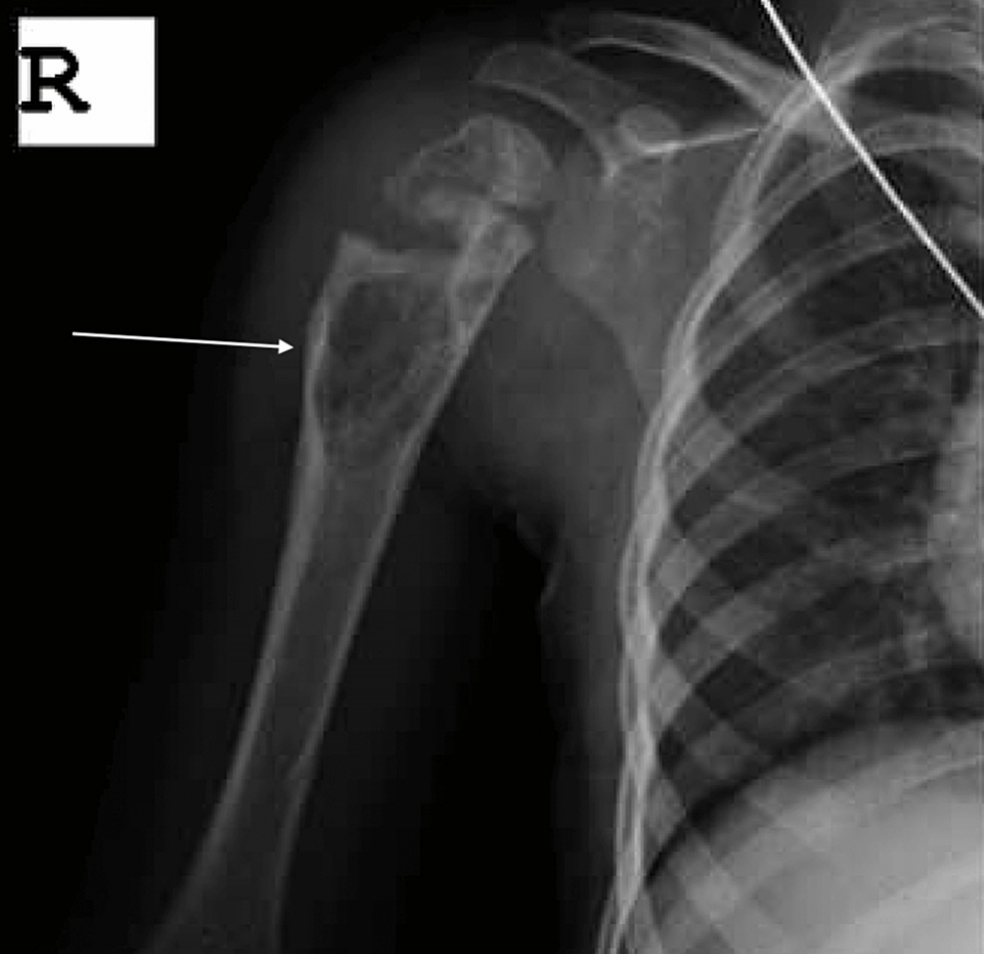
X-ray of the right proximal humerus

Anteroposterior (AP) and lateral X-rays of the right proximal humerus showed a large lucency over the lateral humerus distal to the physis (Figure 1).

**Figure 2:**
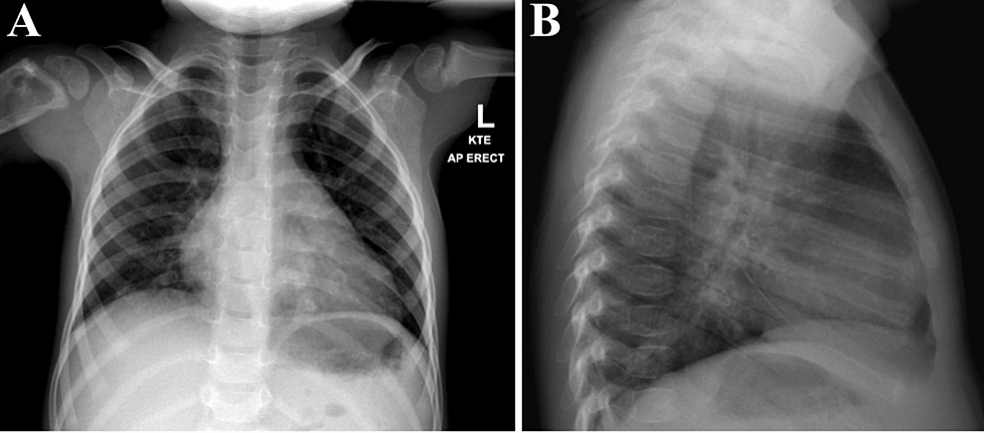
X-ray of the chest in the AP (A) and lateral (B) views AP: Anteroposterior

A chest X-ray showed mild perihilar infiltrates with superimposed patchy infiltrate versus atelectasis in the retrocardiac region (Figure 2).

**Figure 3:**
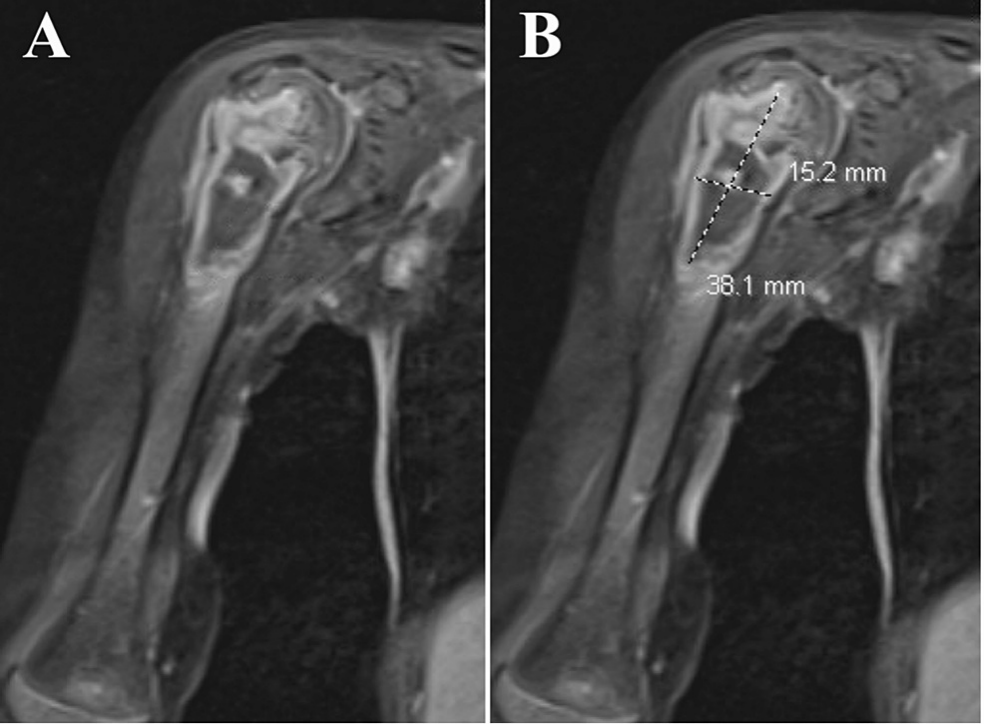
MRI of the right proximal humerus (A) with a Brodie's abscess marked (B)

Chest X-ray findings were inconsistent with an active pulmonary TB infection. MRI revealed proximal humeral osteomyelitis with a Brodie's abscess seen as a 15.2 x 38.1 mm peripherally enhancing fluid collection within the posterior subacromial subdeltoid bursa (Figure 3).

The patient subsequently underwent a computerized tomography (CT)-guided needle biopsy from the shoulder mass and the adjacent soft tissue bursitis/abscess. Approximately ten milliliters of purulent fluid were aspirated, and a core biopsy was obtained to rule out malignancy. The patient also underwent an incision and drainage of the abscess with curettage and debridement of the proximal humerus osteomyelitis and extraction of caseous material from the abscess. The samples were sent for polymerase chain reaction (PCR) testing. Post-operatively, she remained afebrile and without complications.

Diagnosis and treatment

**Figure 4:**
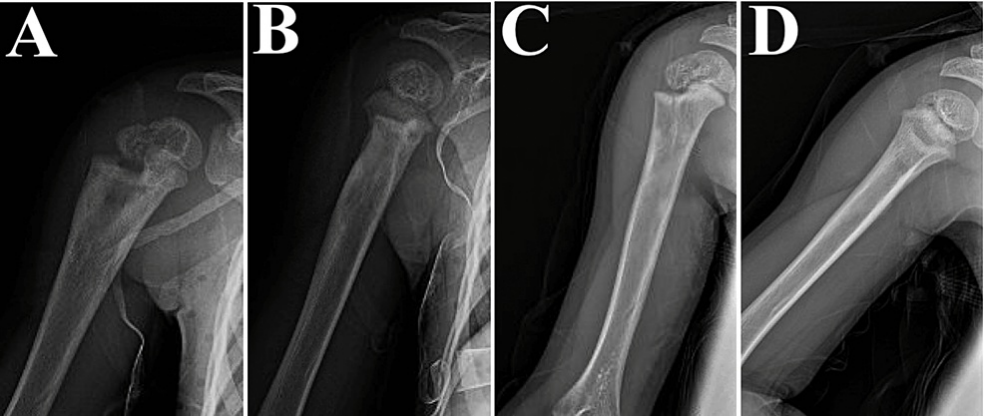
X-rays of the right proximal humerus three months after discharge in AP (A) and lateral (B) views and twelve months after discharge in AP (C) and lateral (D) views AP: Anteroposterior

Due to the indolent nature of bone findings and lack of clinical symptoms, there were concerns for Kingella kingae or TB infections. Additional differential diagnoses were a bone cyst, bone tumor, and Staphylococcal osteomyelitis. The patient was placed on intravenous (IV) ceftriaxone and airborne precautions over infection concerns. Pertinent laboratory tests were ordered. A biopsy of the right humerus showed non-necrotizing granulomata. Acid-fast bacillus (AFB) sputum smears obtained from gastric washes, gram stains, and cultures were negative. However, the purified protein derivative (PPD), QuantiFERON-TB Gold (QFT), and PCR tests of the aforementioned samples were positive for TB. An AFB culture taken from the humeral abscess had growth positive for the Mycobacterium tuberculosis complex as well. The diagnosis of TB was confirmed at this point and the Florida Department of Health was notified. IV ceftriaxone was discontinued following positive TB testing and a TB regimen with rifampin, isoniazid, pyrazinamide, and ethambutol was initiated. Vitamin B6 was given as well. Ethambutol was later switched to levofloxacin for better bone penetration. The patient remained stable with no new signs or symptoms of infection. She was discharged on hospital day nine with follow-up appointments scheduled, including one at the TB division of the Florida Department of Health. Post-discharge, TB GeneXpert results showed drug sensitivity to rifampin and isoniazid. The patient continued her antibiotic treatment post-discharge, with her antibiotic regimen managed by the Florida Department of Health. Follow-up X-rays three and twelve months after diagnosis showed continued lesion healing (Figure 4).

## Discussion

TB osteomyelitis often presents with clinical manifestations due to underlying infection and inflammation including erythema, calor, soft tissue swelling, bone pain, enlarged regional lymph nodes, a draining abscess or sinus, and bony deformity. Constitutional symptoms such as fever, malaise, and night sweats are less common [11]. Our patient had an indolent course with a non-painful bone mass as the only presenting sign of TB infection. This presentation is exceedingly rare, with few case reports of TB osteomyelitis in the proximal humerus or of TB presenting as an asymptomatic bone mass. The patient's presentation highlights the potential for atypical manifestations of TB and suggests that TB should be in the differential diagnosis for bone masses, even in the absence of pulmonary or systemic symptoms.

Of note, the initial AFB smears in gastric washes tested negative in our patient, despite a confirmed diagnosis of TB via PPD, QFT, and PCR tests, highlighting the need for fluid or tissue samples in TB osteomyelitis testing rather than only sputum smears. The recommended treatment for extrapulmonary TB is a nine-month course of antitubercular agents, resulting in complete recovery in 96% of patients [12]. However, TB osteomyelitis has a recovery rate of 87% following a nine-month course of antitubercular agents, so a 12-month treatment regimen is recommended [12]. Timely diagnosis and appropriate treatment are key to effective TB recovery and prevention of complications such as death, meningitis, military TB, or the development of drug resistance.

## Conclusions

TB osteomyelitis can cause extensive local bone destruction. Therefore, timely detection and treatment are necessary to prevent permanent damage and disability. Due to the low prevalence and potential for atypical presentations, diagnosing TB osteomyelitis is challenging even for experienced clinicians. A high index of suspicion should be maintained for patients with risk factors for infection, including time spent in TB-affected regions or close contacts from endemic areas. Our patient presented with a rare manifestation of tuberculosis as an indolent bone mass not commonly seen in developed countries. Had the travel history to Haiti not been ascertained, necessary treatment would have been delayed and the patient may have been subject to unnecessary procedures to determine a diagnosis. This case highlights the importance of obtaining detailed histories, particularly in cases without a clear diagnosis.
